# Identification and validation of fusidic acid and flufenamic acid as inhibitors of SARS-CoV-2 replication using DrugSolver CavitomiX

**DOI:** 10.1038/s41598-023-39071-z

**Published:** 2023-07-21

**Authors:** M. Hetmann, C. Langner, V. Durmaz, M. Cespugli, K. Köchl, A. Krassnigg, K. Blaschitz, S. Groiss, M. Loibner, D. Ruau, K. Zatloukal, K. Gruber, G. Steinkellner, C. C. Gruber

**Affiliations:** 1 Innophore, San Francisco, CA USA; 2 Institute of Molecular Biosciences, University of Graz, Graz, Austria; 3 Austrian Centre of Industrial Biotechnology, Graz, Austria; 4 Field of Excellence BioHealth - University of Graz, Graz, Austria; 5 Diagnostic and Research Institute of Pathology, Medical University of Graz, Graz, Austria; 6 NVIDIA, Santa Clara, CA USA

**Keywords:** Drug development, Viral proteins, Computational science

## Abstract

In this work, we present DrugSolver CavitomiX, a novel computational pipeline for drug repurposing and identifying ligands and inhibitors of target enzymes. The pipeline is based on cavity point clouds representing physico-chemical properties of the cavity induced solely by the protein. To test the pipeline’s ability to identify inhibitors, we chose enzymes essential for SARS-CoV-2 replication as a test system. The active-site cavities of the viral enzymes *main protease* (M^pro^) and *papain-like protease* (Pl^pro^), as well as of the human transmembrane serine protease 2 (TMPRSS2), were selected as target cavities. Using active-site point-cloud comparisons, it was possible to identify two compounds—flufenamic acid and fusidic acid—which show strong inhibition of viral replication. The complexes from which fusidic acid and flufenamic acid were derived would not have been identified using classical sequence- and structure-based methods as they show very little structural (TM-score: 0.1 and 0.09, respectively) and very low sequence (~ 5%) identity to M^pro^ and TMPRSS2, respectively. Furthermore, a cavity-based off-target screening was performed using acetylcholinesterase (AChE) as an example. Using cavity comparisons, the human carboxylesterase was successfully identified, which is a described off-target for AChE inhibitors.

## Introduction

The comparison of biomolecular active sites is an emerging method to predict enzymatic function and potential ligands^1^. Especially since AI-based structure predictions^2–4^ now provide access to billions of novel protein structures, the annotation, interpretation, and comparison of binding sites has become an essential part of everyday bioinformatics. In this work, we examine the potential of the Catalophore™ technology^5,6^ to predict possible inhibitors for selected enzymes using Catalophores. In short, Catalophores are 3D multivariate point clouds representing the spatial distribution of a set of physico-chemical properties including, among others, hydrophobicity and electrostatics.

Recently, Catalophores have been used in the prediction of the binding affinity of human angiotensin-converting enzyme 2 (hACE2) to the receptor-binding domain (RBD) of SARS-CoV-2 spike protein^7^, to track changes in the binding interface of SARS-CoV-2 spike variants^8^, and for protein engineering^9^.

Catalophores enable the study of enzymes beyond the structural level. They allow an analysis based on cavities of active sites represented by 3D point clouds. Representing cavities and their properties as 3D point clouds has a great advantage in the usage of machine-learning methods, where it corresponds to voxelization. This technology “makes biology machine-readable”, and we refer to it as *CavitomiX*.

Herein we report a CavitomiX-based pipeline for drug repurposing and inhibitor search. In this method, Catalophore point clouds are compared by an iterative-closest-point matching algorithm. It is important to understand that this comparison is independent of sequence- or structural identity.

**Figure 1:**
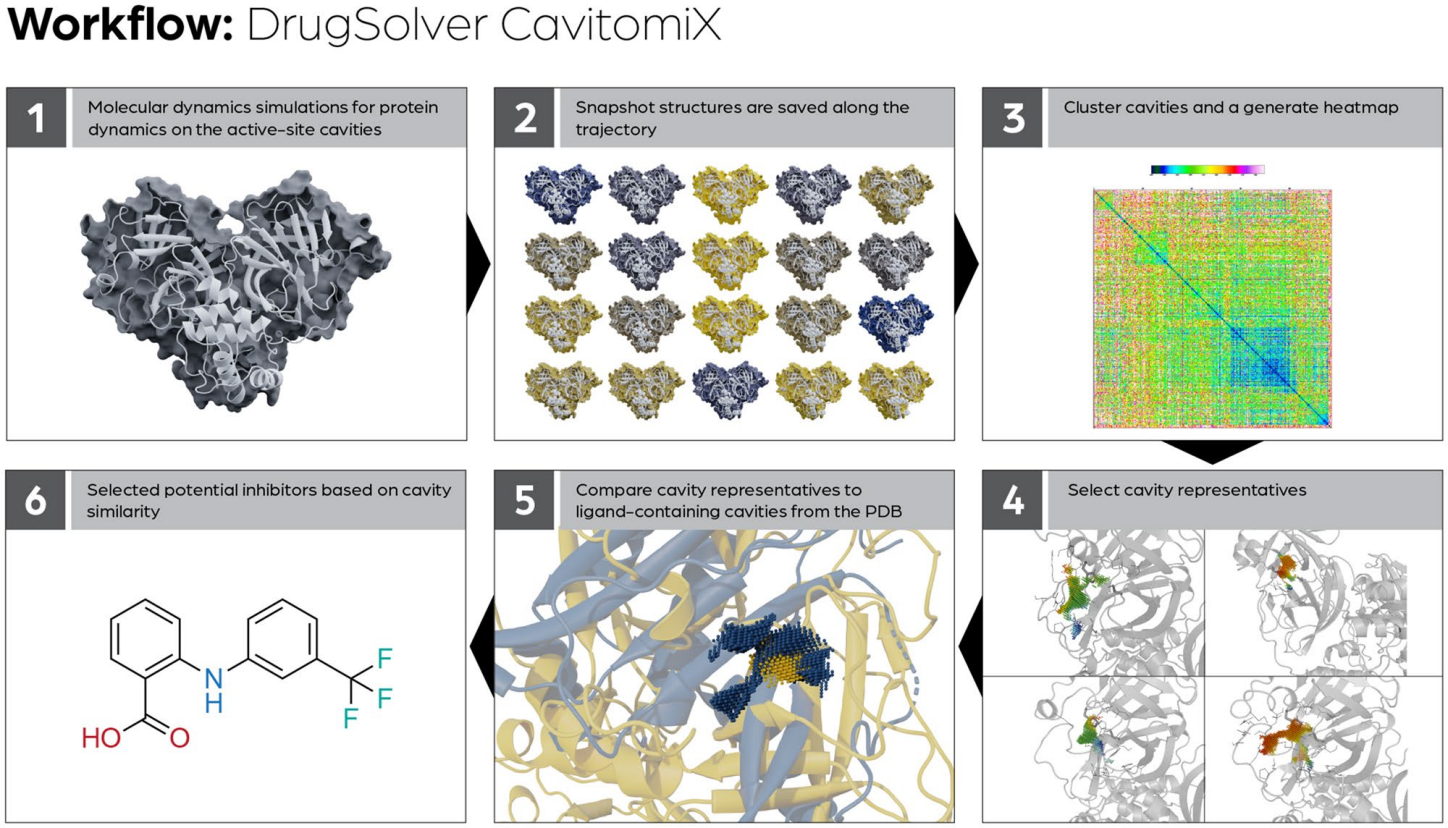
General description of the DrugSolver CavitomiX workflow to identify inhibitors based on active-site cavity similarity.

In Fig. 1, an overview of the workflow is shown: Starting from the models of the structures, molecular dynamics (MD) simulations are performed to address the influence of protein dynamics on the active-site cavities. Snapshot structures are saved along the trajectory. In these snapshot structures, the cavities are detected automatically, and the active-site cavities are selected. (A cavity is chosen as an active-site cavity if it aligns the active-site residues of the enzyme).

To reduce the number of cavities used for comparisons against cavities from the Protein Data Bank (PDB), all active-site cavities calculated in the snapshot structures are compared to each other. Based on this comparison, the cavities are clustered, and a heatmap is calculated. For the larger clusters, cavity representatives are selected. These cavities represent a specific dynamic state of the cavity observed in the trajectory. The cavity representatives are then compared to a dataset of ligand-containing cavities (e.g., with cavities of ligand-containing proteins from the PDB). The ligands of the most similar cavities to the cavity of the target enzyme are selected as potential inhibitors.

We tested the ability of DrugSolver CavitomiX as a tool for drug repurposing for the case of SARS-CoV-2 to identify possible inhibitors of the SARS-CoV-2 M^pro^, the SARS-CoV-2 Pl^pro^, and the host TMPRSS2, all enzymes essential for viral replication. In the following, we briefly describe these three proteins and their role. Viral cell entry depends on the binding of the S (Spike) protein to the human ACE2 receptor^10^. The S1-domain of the spike protein binds to the receptor. In addition to that, a process called "S-priming" is necessary. Here the S protein is cleaved at the S1/S2 site, which enables membrane fusion—a process driven by the S2 subunit^11^. The human transmembrane protease serine 2 (TMPRSS2) is used for S protein priming^12–14^.

Consequently, inhibition of this protease could result in decreased virus uptake into the cell. Therefore, this enzyme is an interesting drug target. After cell entry, the non-structural proteins (NSPs) of SARS-CoV-2 are responsible for viral replication in human cells. One of the essential NSPs is the 3Cl-like protease or main protease (M^pro^).

M^pro^ is a cysteine protease and employs a catalytic diad, which consists of a cysteine and a histidine. Cysteine proteases can also employ a catalytic triad containing glutamine, glutamate, asparagine, or aspartate as the third catalytic residues. In the case of the M^pro^, a water molecule takes over the role of this third residue^15–17^. M^pro^ is an attractive drug target because it is essential to cleave the coronavirus polyprotein at 11 cleavage sites downstream of NSP4^18–20^. This function makes it a crucial enzyme for processing non-structural proteins. Essential viral enzymes cannot be active without further proteolytic release^21^. Thus, M^pro^ is a key enzyme for viral replication^18–20^.

Furthermore, its substrate specificity—M^pro^ only cleaves polyprotein sequences after a glutamine residue—is interesting for a drug target since no human protease is known with this substrate specificity. This could lower the potential side effects of a drug targeting M^pro^^22–25^.

The papain-like protease (Pl^pro^) is the second viral protease. Like M^pro^, Pl^pro^ is a cysteine protease. Its catalytic triad consists of Asp, His, and Cys. It plays a role in polyprotein processing and is essential for viral replication. It cleaves NSP1, NSP2, and NSP3 at the recognition site ”LXGG↓XX”^26,27^. This is essential for the maturation of the polyprotein and makes Pl^pro^ a promising drug target. The predicted inhibitors were tested for their ability to inhibit the target enzymes in wet-lab experiments using a Caco-2 cell-based viral-infection assay.

In the second validation experiment, we tested the ability of the Catalophore™ based DrugSolver CavitomiX platform to identify off-targets. For this application, the example of acetylcholinesterase (AChE) was chosen. AChE is an essential enzyme in the central nervous system of animals and the target for drugs against Alzheimer’s disease (AD)^28^ as well as the target of the highly potent organophosphorus nerve agents, like Sarin or Novichok^29^. AChE terminates neurotransmission at cholinergic synapses by hydrolyzing acetylcholine (ACh), consequently regulating the neurotransmitter’s concentration^30^. In AD treatment, that process is slowed down through inhibitors to enhance the low concentrations of ACh in the synaptic cleft. The Novichok agents, on the other hand, covalently bind to the catalytic triad of AChE and thus permanently inactivates AChE, leading to lethal levels of ACh accumulation^29^. The identification of off-targets is highly interesting since it helps to predict and potentially avoid possible side effects of AD drugs or harmful effects of nerve agents. Off-targets are identified by the similarity of their binding-site cavities to the active-site cavity of AChE. To this end, a search was performed in an uncurated dataset of cavities of PDB-proteins (i.e. all cavities from PDB proteins, calculated with standard parameters, without any manual selection or curation).

## Results

### Calculated active-site cavities and cavity representatives for different dynamic states

**Figure 2:**
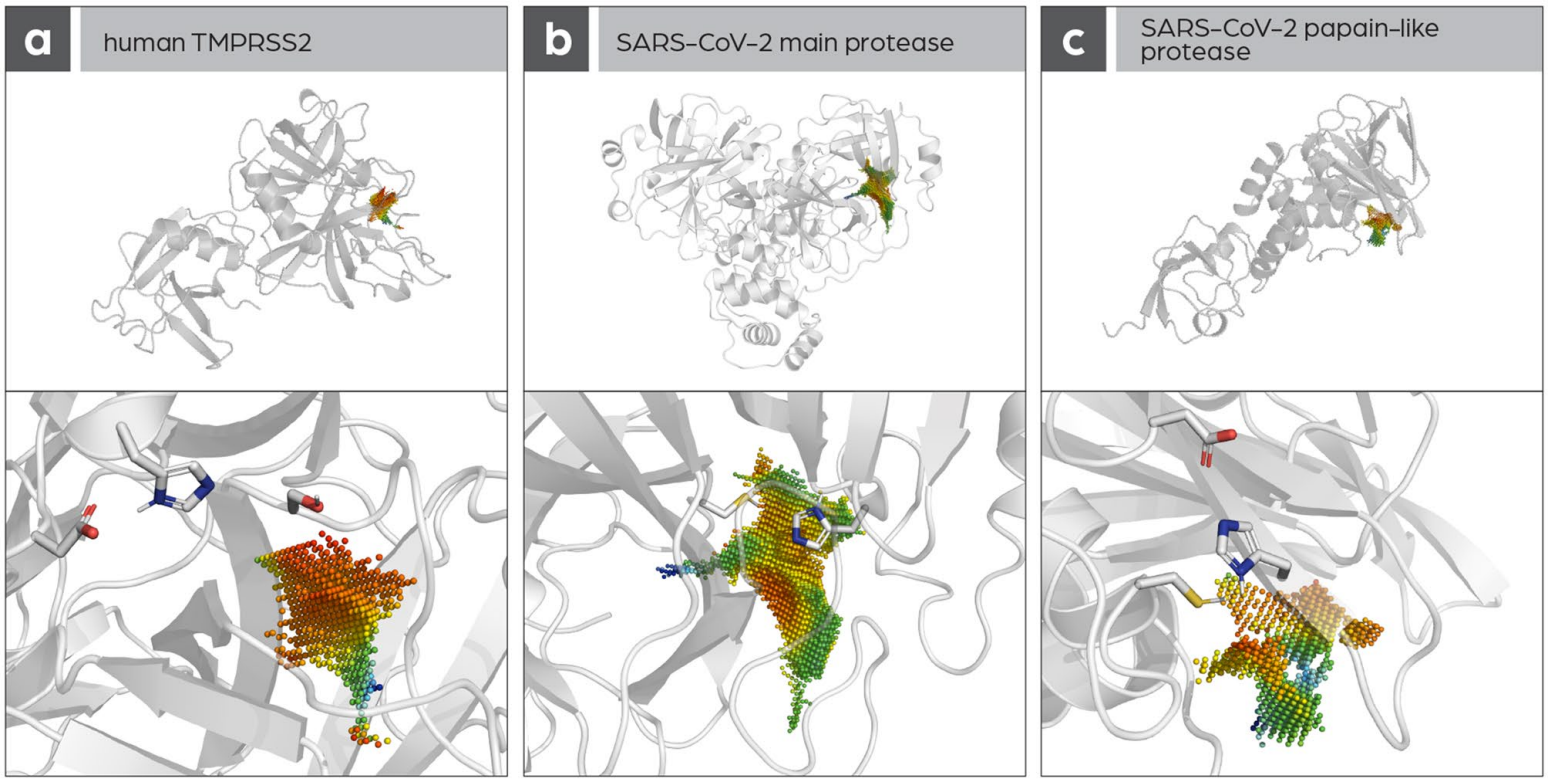
Active-site cavities in (**A**) human TMPRSS2, (**B**) SARS-CoV-2 M^pro^, and (**C**) SARS-CoV-2 Pl^pro^.

First all detectable cavities of the main protease, TMPRSS2, and papain-like protease were calculated; the Catalophore™ technology^5,6^ makes it possible to represent cavities as 3D-point clouds and attribute a set of physico-chemical properties to each cavity point. After that, the cavity aligning the active-site residues was selected as the active-site cavity. (as shown in Fig. 2). Then, MD simulations were carried out: 10 ns (TMPRSS2, PI^Pro^) and 100 µs (M^pro^). 1000 snapshot structures per protein were created along the trajectory and the active-site cavities were calculated in all these structures. Using hierarchical clustering methods all the resulting active-site cavities were grouped into clusters based on point cloud similarities. These clusters can be interpreted to represent different states of the cavities created by protein dynamics. For each larger cluster, a cavity representative was calculated and used in an additional set of cavity comparisons. This way, protein dynamics and their influence on protein binding pockets can be considered.

### Cavity-based identification of potential inhibitors

As mentioned above, the following strategy was used to identify potential inhibitors for M^pro^, Pl^pro^, and TMPRSS2: similar cavities to the calculated active-site cavity representatives with bound ligands were identified. Enzymes with a similar active site should show similar small-molecule-binding properties. The calculated cavity representatives were compared with a set of cavities from the PDB with the condition that the cavity must contain a co-crystallized ligand. This way, it was possible to gather a dataset of approximately 100,000 cavities with a known binding partner to which the cavity cluster representatives could be compared. The ligands of the most similar cavities to the target cavities were selected for testing their inhibitory effect on SARS-CoV-2 replication.

### Potential inhibitors identified for TMPRSS2 and the SARS-CoV-2 protease

**Figure 3:**
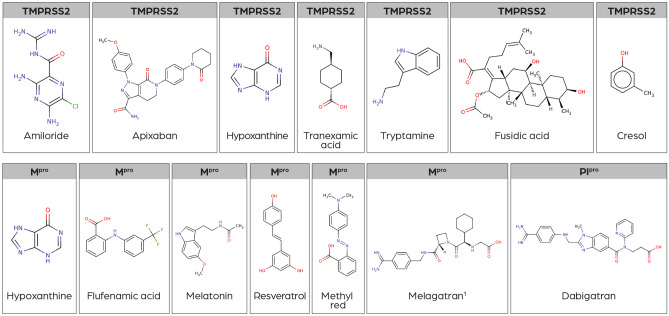
Structures of potential inhibitors identified by the cavity-based workflow. (^1^At the time of this work, melagatran was not available to purchase at Sigma-Aldrich; therefore, the prodrug ximelagatran (the alkylated version of melagatran) was ordered and tested instead).

The following compounds (shown in Fig. 3) were identified as potential inhibitors for the SARS-CoV-2 M^pro^, SARS-CoV-2 Pl^pro^, and the human TMPRSS2 by the high similarity of the compound-binding cavities to the active-site cavities of M^pro^/Pl^pro^/TMPRSS2.

### Cell-based virus replication assay test to determine the inhibitory effects of selected compounds on the replication of SARS-CoV-2

**Figure 4:**
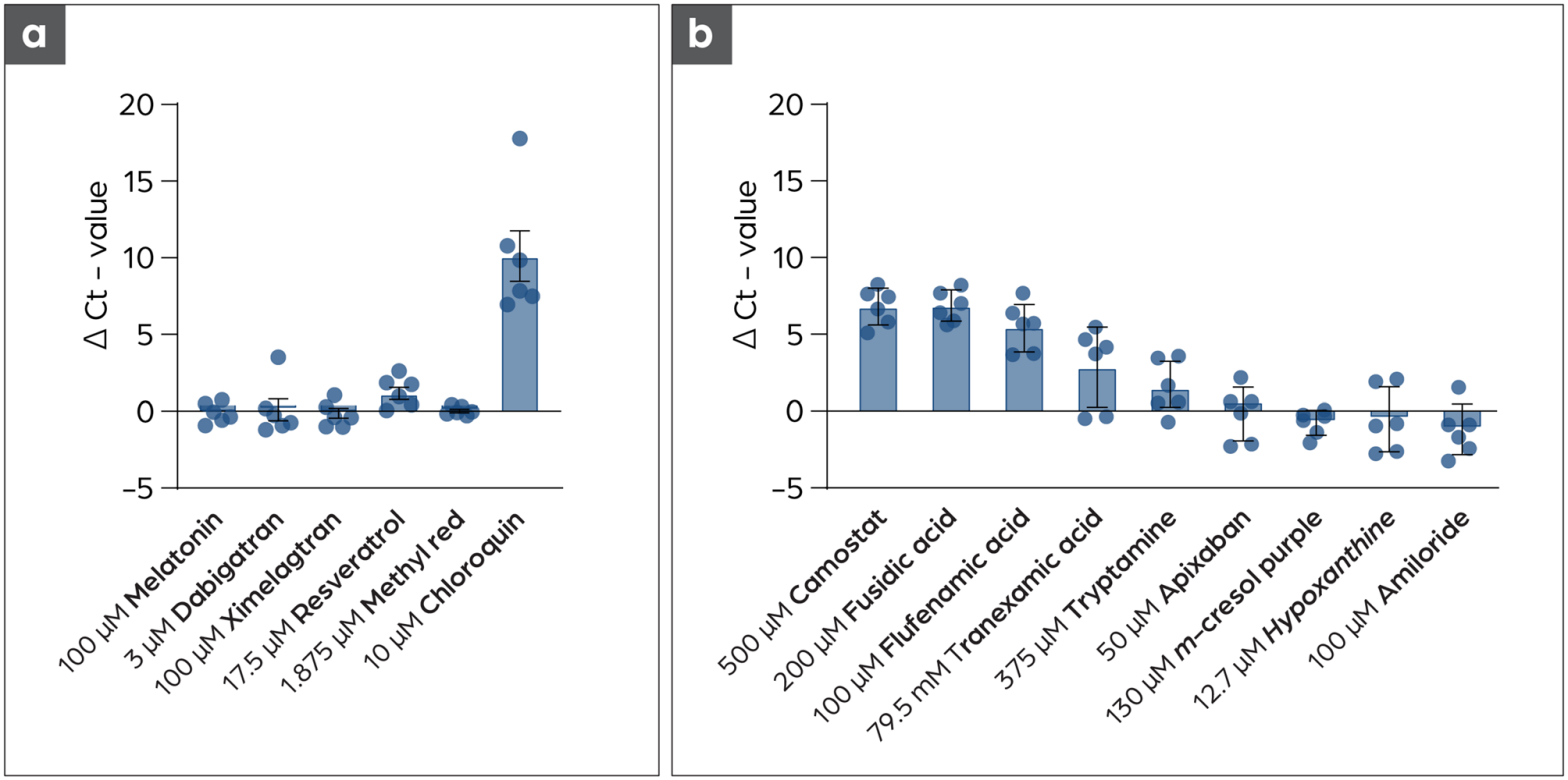
Testing of putative inhibitors in cell-based based virus replication assays. (**a**) None of the tested substances show an antiviral effect against SARS-CoV-2 on Vero CCL81 cells. Substances were added for ½ h before infection of each well with 12 PFU SARS-CoV-2. The cell culture supernatant was harvested after 48 h, followed by RNA-extraction and qRT-PCR for the nucleocapsid target (N1 primer). Chloroquine was used as a control substance^31,32^. ∆Ct values were calculated for better comparability by subtracting the Ct-value of the infected samples without substance pre-treatment from the infected samples with substance pre-treatment n = 6. (**b**) Fusidic acid and flufenamic acid show inhibition of SARS-CoV-2 replication on Caco-2 cells. Cells were pretreated with the respective substance for 1/2 h, followed by SARS-CoV-2 infection (MOI: 0.825). The supernatant was harvested after 48 h followed by RNA extraction and qRT-PCR analysis (N2 primer).

First compounds targeted against the M^pro^ were tested on Vero CCL81 cells at the highest concentration where no cytotoxic effect were observed (Fig. S1). None of the tested substances showed a strong antiviral effect, except chloroquine, which was used as a control substance as it is known to inhibit viral replication in Vero cells^31^ despite not showing therapeutic benefit in clinical trials^33^. Only low antiviral activity was found for resveratrol. This substance is known to bind unspecifically to many proteins and influences a broad variety of cellular factors. Binding to the M^pro^ cannot be excluded, but many other factors may also be influenced by resveratrol and result in the low antiviral effect observed^34^. In parallel, a cell-based virus replication assay suitable to test TMPRSS2 inhibitors was established. A Caco-2 cell-based assay was chosen since Caco-2 cells were shown to express TMPRSS2 (Fig. S4). This assay was used to test potential inhibitors for TMPRSS2 and also for testing potential inhibitors of M^pro^ and Pl^pro^, which were not tested in the first round using the Vero cell-based assay. Caco-2 cells were infected at a high viral load (MOI: 0.825) to identify a strong antiviral effect of the compounds and to exclude possible false-positive results. Samples were taken after 48 h and analyzed with qRT-PCR for the quantification of viral replication^35,36^. Two compounds, fusidic acid and flufenamic acid, show a antiviral effect comparable with the known TMPRSS2 inhibitor Camostat Mesylate as shown in Fig. 4. Additionally, the antiviral effect was confirmed using immunohistochemistry staining of the infected cells. In this semiquantitative assay, infected cells were visualized utilizing a SARS-CoV-2 antibody directed against the nucleocapsid protein (N). As shown in Fig. S3, fewer cells got infected compared to an infected control when treated with fusidic acid or flufenamic acid during the assay and incubation period.

### Fusidic acid and flufenamic acid were identified as inhibitors of viral replication

#### Fusidic acid

**Figure 5:**
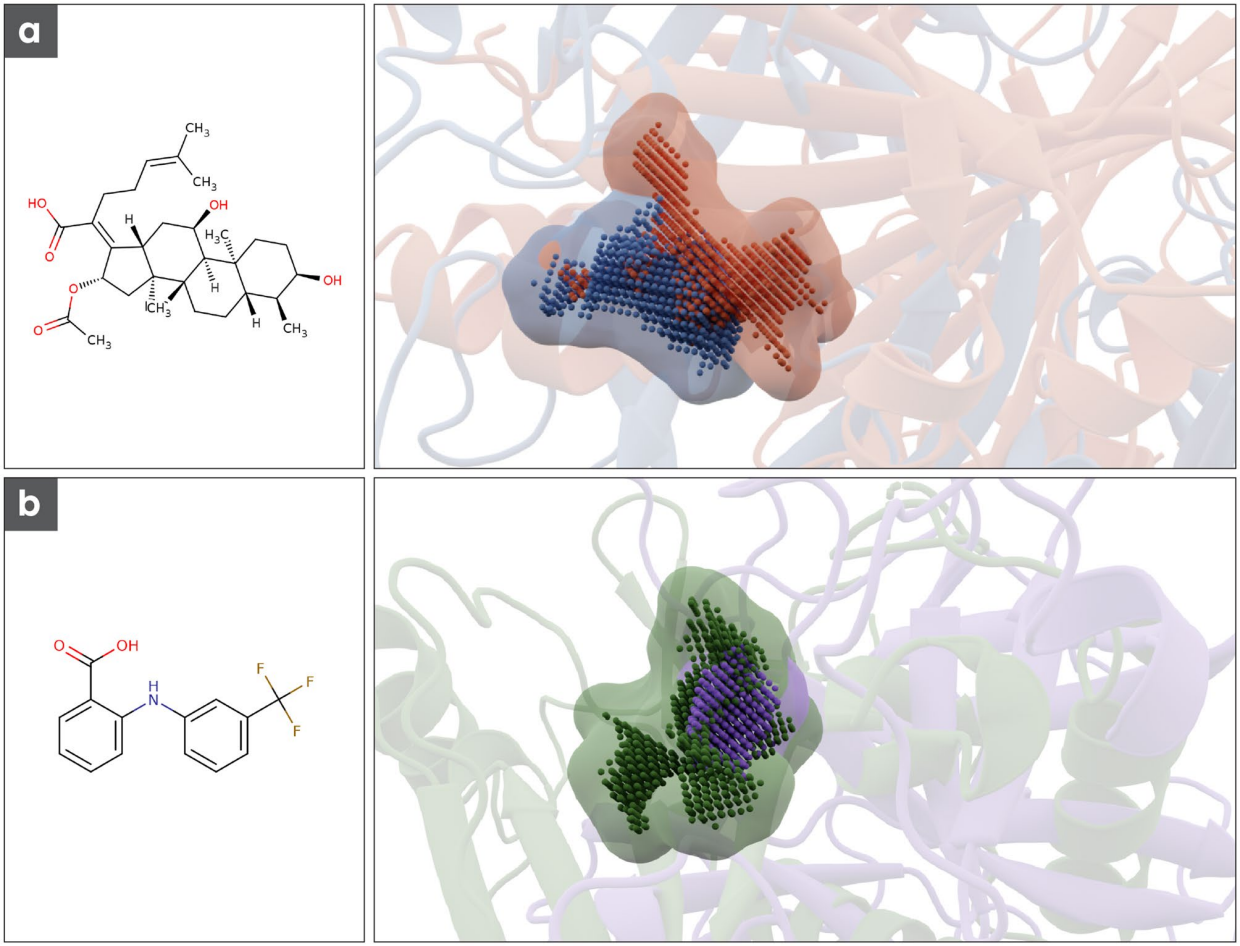
(**a**) Chemical structure of fusidic acid and the corresponding cavity match. In blue structure and cavity of the M^pro^ in red transcription factor TEAD2 (**b**) Chemical structure of flufenamic acid and the corresponding cavity match. In purple structure and cavity of TMPRSS2 in purple Chloramphenicol acetyltransferase.

Active-site cavity representatives of TMPRSS2 show a high similarity to the ligand-binding cavity of the *Escherichia coli* enzyme chloramphenicol acetyltransferase 3 (PDB-ID: 1QCA). The corresponding cavity match can be seen in Fig. 5a. Chloramphenicol acetyltransferase and TMPRSS2 show no significant sequence identity (~ 5%) or structural similarity (Tm score: 0.09). Fusidic acid is a potent competitive inhibitor of chloramphenicol acetyltransferase 3^37^ and is an approved drug that has been used as an antibiotic since the 1960s^38^, making it an attractive candidate as a potential inhibitor of TMPRSS2.

#### Flufenamic acid

The active-site cavity representatives of the M^pro^ show a high similarity to a cavity of transcription factor TEAD2 with bound flufenamic acid (Fig. 5b). The main protease and the transcription factor TEAD2 are sequentially and structurally diverse and therefore show a very low sequence identity of about 4% and a very low Tm score of 0.1. Flufenamic acid was invented and approved in the 1960s^39^ and is mainly used as an analgesic drug in treating the pain associated with rheumatoid diseases^40^.

### Cavity-based identification of CSE1 as an off-target for AChE inhibitors

**Figure 6:**
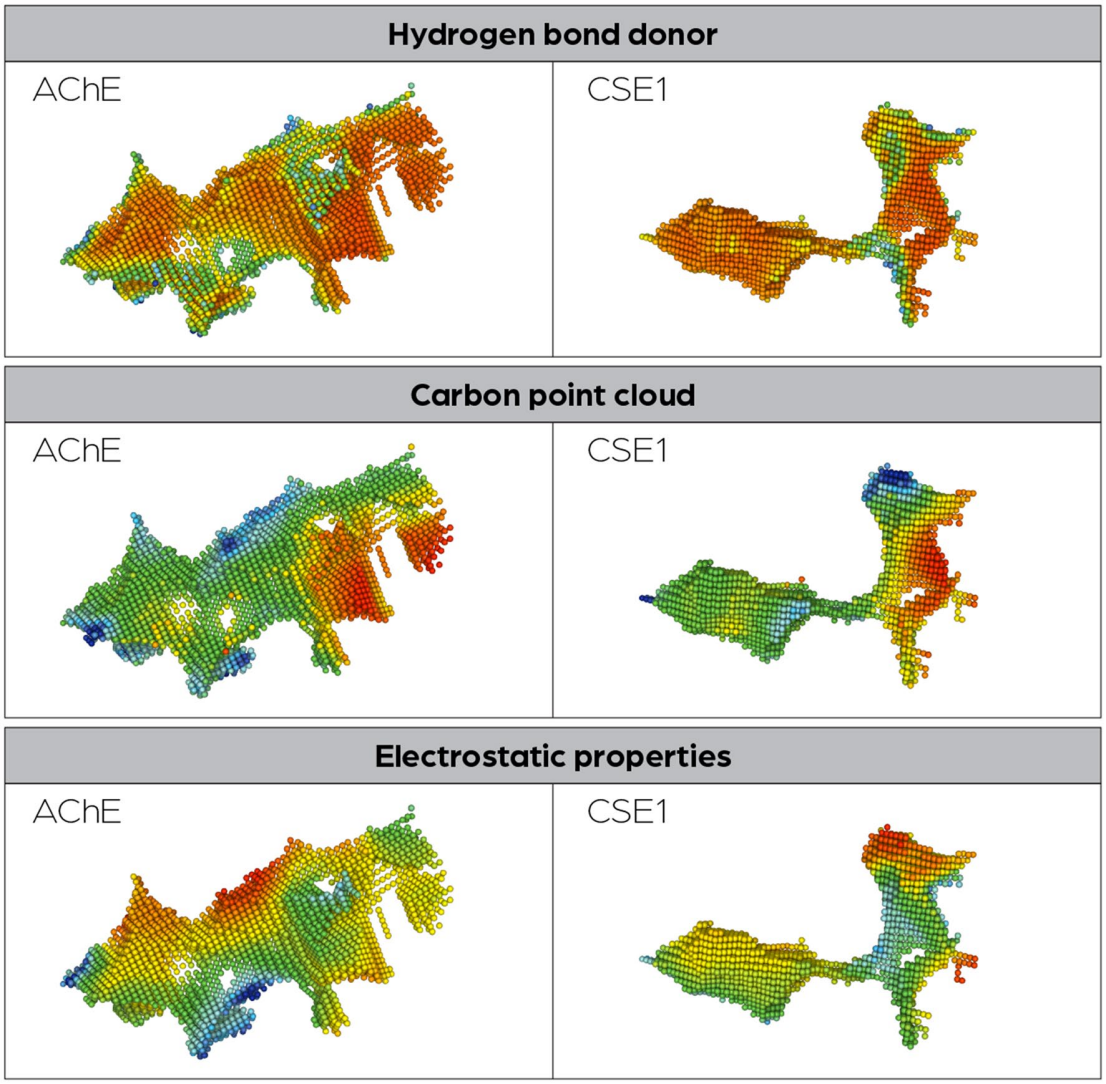
Side-by-side comparison of active-site cavities from AChE and CSE1.

The cavity-comparison results were ranked by similarity to the acute-site cavity of AChE. Among the top hits, human carboxylesterase 1 (CES1) was identified (PDB-ID: 5a7h), the active-site cavities of AChE and CSE1 are shown in Fig. 6. CES1 is known to bind AChE inhibitors, making it an off-target for drugs targeting AChE. The fact that it can be identified using the Catalophore™ DrugSolver platform shows that also off-target identification is possible, despite the actual geometric shape of the cavities, which were identified to have similar properties, differ significantly.

## Discussion

In this work, we established a workflow based on active site cavity comparisons, capable of identifying inhibitors for selected target enzymes. This technology enables a novel approach towards drug repurposing since it is independent of structure and sequence alignments. DrugSolver CavitomiX delivered two already approved drugs that show anti-viral activity against SARS-CoV-2 in infection assays: fusidic and flufenamic acid. These two compounds were found as proteins binding them had similar cavities as our target enzymes. Despite their high active-site cavity similarity, they show very little structural (TM-score^41^ of 0.09 and 0.1) and very low sequence (around 5%) identity to M^pro^ and TMPRSS2, respectively. Thus, these two proteins (and consequently their ligands) would never have been identified using sequence- and structure-based comparisons.

Now that the computational pipeline is established, it can quickly be modified and adapted to address new pathogens as needed. In future versions, a combination with technologies such as DrugSolver pathogen-seqscan^42^, which enables the automatic detection of druggable targets in new pathogens and is part of Innophore’s DrugSolver platform, makes it possible to address newly occurring pathogens even faster. Also, in the case of mutating drug targets, like SARS-CoV-2 M^pro^^43^, the mutated targets can quickly be re-addressed if needed.

In the last years, more and more efforts have been devoted to the development of novel methods of protein structure prediction. Methods like AlphaFold2^2^, RoseTTAFold^4^, ESM fold^3^, and the BioNeMo toolbox (https://www.nvidia.com/en-us/gpu-cloud/bionemo/) were made available to the public, and follow-up versions of these methods are currently in development. These new methods, among other drug repurposing approaches^44^, make DrugSolver CavitomiX even more potent. It enables its users to quickly predict the proteome of newly emerging pathogens. Using the Catalophore™ CavitomiX approach, the cavitome of new pathogens can be calculated, and the here-described workflow enables the rapid identification of potential inhibitors.

CES1 is already described to be a target of AChE inhibitors. The enzyme was even suggested to be applied as a bio-scavenger to protect against organophosphorus nerve agents, which are known to bind to AChE^45^. Finding the CES1 among the best hits in the Catalophore™ workflow is a promising result, showing the potential of the Catalophore™ DrugSolver CavitomiX to be used for off-target identification. A combination of drug identification and off-target identification in one workflow has great potential. Identifying off-targets is of great interest for predicting and preventing side effects. The given example here serves as a proof-of-principle, and the off-target identification using 3D cavity property point cloud information will be further improved in future work.

## Methods

### Homology model

For TMPRSS2 and Pl^pro^, SWISS-models^46–49^ were built using default settings. While Pl^pro^ had an 83% sequence identity with its crystal template (PDB-ID: 5y3e), the best template for TMPRSS2 revealed only 38% identity with serine protease Hepsin (PDB-ID: 5ce1) automatically selected as a template. For the main protease, we used the model as well as MD-simulation data of the D.E. Shaw research center, which had been published in March 2020^50^.

### Molecular dynamics simulations

Own MD simulations have been carried out using Yasara version 20.4.24^51^ with the Amber03 force field. For each system, 1000 structural snapshots with a constant time interval of 10 ps in between had been sampled, covering a total time range of 10 ns. However, regarding M^pro^ MD simulations of 100 µs length, run on Anton supercomputers at D.E. Shaw research, the 1000 extracted snapshots had 100 ns intervals in between. Snapshots of all systems were used for the next workflow steps.

### Calculation of cavity point clouds

**Figure 7:**
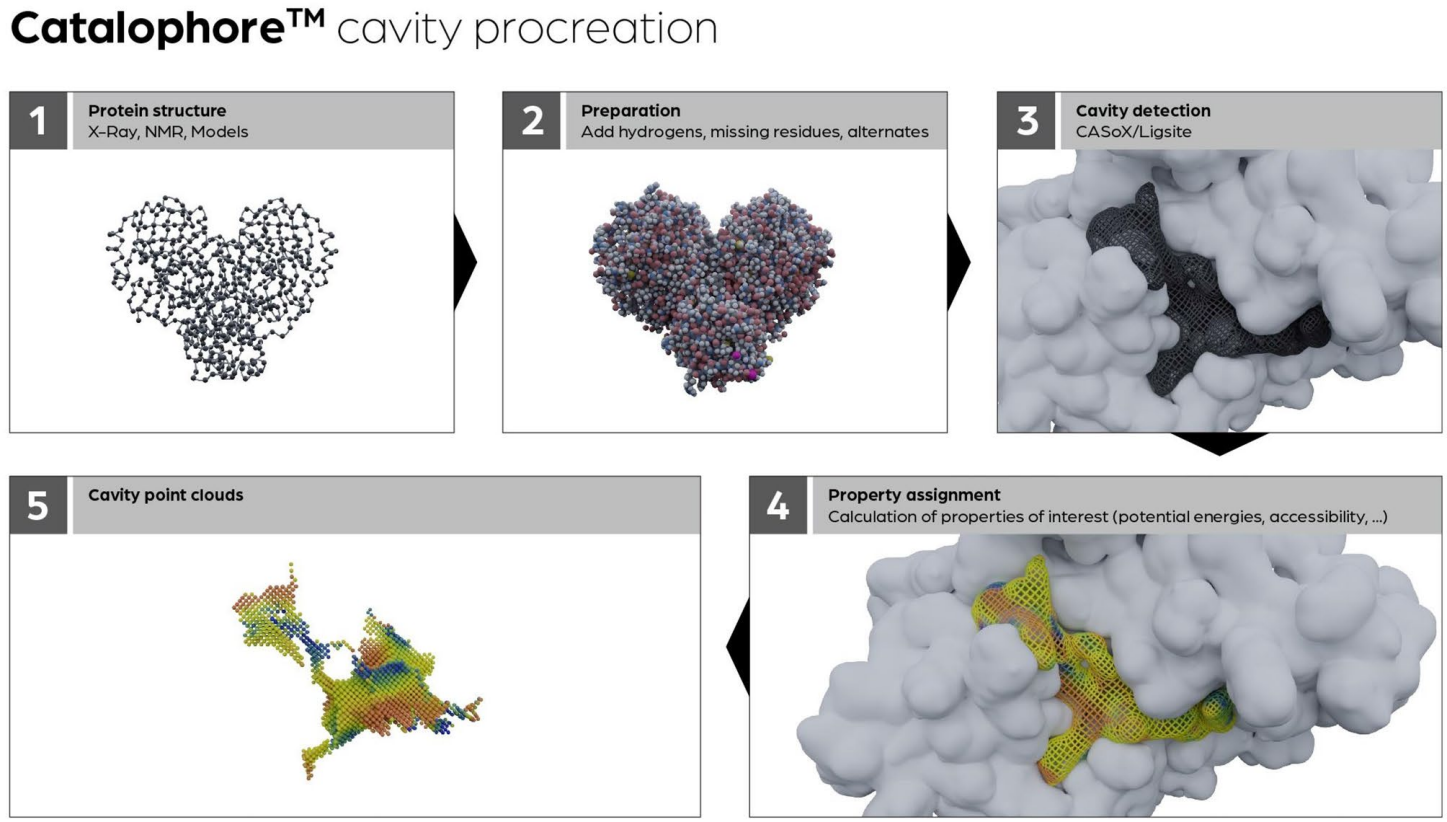
Catalophore™ cavity procreation workflow scheme.

For the calculation of the cavity point clouds, the Catalophore™ technology^5,6^ is used. The workflow scheme to calculate protein cavities is shown in Fig. 7. The cavity calculation is based on a protein structure. This can be an experimental structure, a homology model, or a structure derived from an MD simulation. The first step is the preparation step: here, hydrogen atoms and potentially missing residues are added, and alternates are handled. For cavity detection, the ligsite algorithm^52^ is used. The algorithm searches for protein-non-protein–protein events on each grid point in a line on the axes. The analysis of the grid points helps identify closed cavities and points that are accessible to the solvent.

Grid settings are important to find the same cavities with different orientations in space, and the defined axis directions are usually sufficient to determine cavities appropriately. Additionally, a softness approach is employed to compensate for slight variations in grid settings and eliminate small surface cavities without altering the grid spacing or increasing computation time. After detection, a set of 19 properties using AutoGrid4^53^ is assigned to the cavity points. These properties are derived from the three-dimensional arrangement of the amino acids and special surroundings like cofactors and or ligands within the 3D structure. For each physio-chemical property, one property point cloud is calculated. The result is a collection of pooled cavity grid points, each assigned with physico-chemical properties and additional derived values like hydrophobicity and accessibility values. These points can be compared and aligned with cavity point clouds from other proteins based on their individual physico-chemical properties.

### Comparison of two cavities

For the comparison of two cavities, the point clouds from the query and select point clouds from a pre-computed database are matched. More precisely, the point clouds are superimposed and the superposition optimized. To achieve this, differences are evaluated by comparing the closest grid points between the target and the template.

For each property point-cloud pair, a “property score” is generated, representing the quality of the match. A set of weights allows for fine-tuning the influence of the different grid maps (for instance, setting the scoring factor for the H-bond point clouds to zero causes this interaction to be ignored). This scoring is combined with a geometric match into a “total score” and an iterative-closest-point algorithm is performed to optimize it and determine its final value.

### Clustering and identification of cavity-cluster representatives

As described above, we ran MD simulations in order to encode the dynamics of the entire protein and, in particular, the dynamics of the active-site cavity. During each simulation, we created 1000 snapshots of each corresponding MD trajectory. The active-site point clouds were calculated from each snapshot like they would be from the origin protein structure.

**Figure 8:**
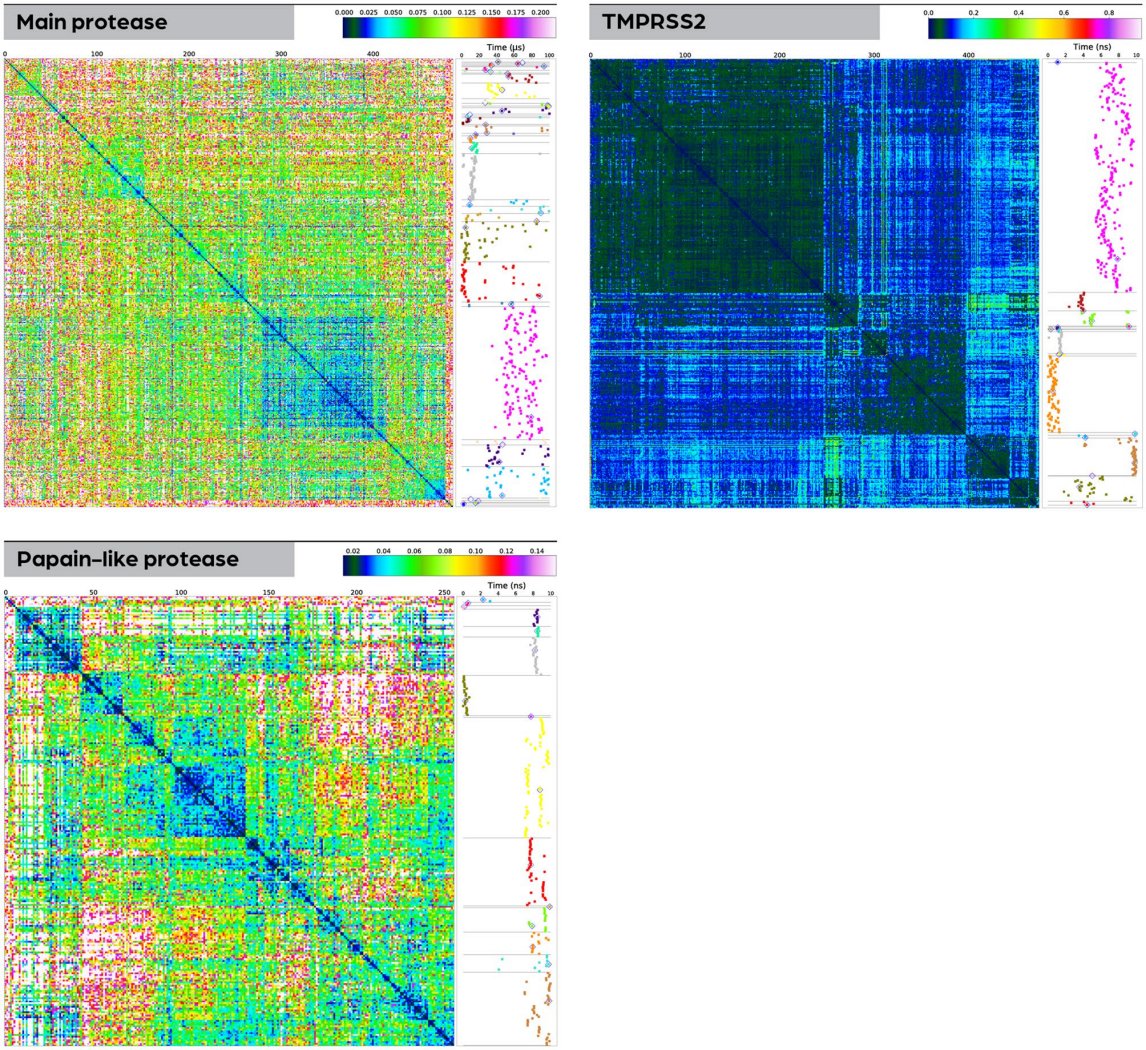
Heatmaps based on clustering of snapshot cavities for the investigated enzymes. Using the clustering, cavity representatives for different dynamic states of the cavity were detected. The heatmap for each molecule (plotted on the left side) shows matching scores of 3D point clouds with the color legend shown above each subfigure. The narrow vertical panel on the right of each heat map contains a time-resolved visualization of cluster assignments for each snapshot’s active-site point cloud. The coloring in this panel is not related to the color scheme in the heatmap but simply indicates the different clusters. The horizontal light gray lines indicate cluster boundaries and correspond to the boundaries’ location in the heatmap. While only representatives of clusters containing more than 5% of all cavities were used further in the workflow, the representatives of all clusters are marked in this figure by a light gray diamond.

This procedure leads to a set of 1000 point clouds representing the active-site-cavity snapshots for each protein. In order to identify different representatives of the dynamic states of the active-site cavities, we compared the point clouds from all snapshots via an all-to-all match. The results from such a matrix match are visualized in the heatmaps in Fig. 8. The identification of dynamical representatives was carried out via hierarchical clustering based on the total-score difference matrix. In fact, the heatmaps in Fig. 8 are already ordered based on the clustering results.

As for choosing the representatives, we limited ourselves to the largest clusters, in particular those that contained more than 5% of the total number of point clouds. This emphasizes those states that are present for large enough amounts of time during the simulation in order to warrant further study. The clusters are illustrated in the side elements next to the heatmaps in Fig. 8, where snapshot location in MD-simulation time is plotted versus the clustering, each cluster is colored differently, and each representative is marked by a diamond (irrespective of cluster size).

A cluster representative was calculated for each large cluster via a minimal-distance requirement to all other cluster members. These representatives were used further in the following steps of the workflow.

### Selection of potential inhibitors

Following the comparison of the cavity datasets, a selection process was undertaken to identify the most promising potential inhibitors. Each cavity in the datasets is assigned a similarity score, reflecting its resemblance to the query cavity. These similarity scores were then utilized to rank the cavities, enabling the identification of the most closely aligned cavities to the query cavity. Additionally, to ensure that only significant results for potential inhibitor binding were considered, cloud-overlap cut-offs (cloud overlap 1 and 2) were considered. These cut-offs, set at 75 and 50 percent, quantified the extent of overlap between two-point clouds during comparison. By applying these criteria, only the most relevant results were retained. Furthermore, cavities containing bound small molecules present in the crystallization conditions were discarded from further analysis. For example, small molecules like polyethylene glycol were considered irrelevant to the investigation and were therefore excluded. Following these steps, the ligands associated with the most similar cavities were investigated and carefully selected for further examination. The availability and accessibility of small molecules were taken into account, ensuring that only those small molecules that could be readily purchased for further experimentation were considered.

### Cytotoxicity assay

To determine the cytotoxic effect of the substances used, Caco2 cells were seeded in 96 well plates at a density of 5000 cells/well in Gibco™ Minimum Essential Medium (MEM with 10% FCS, 1% Pen-Strep) and incubated for 48 h at 37 °C and 5% CO_2_. The medium was then removed, and the respective substance, diluted first in EtOH, then in the respective medium (EtOH concentration was 1% in all conditions), was added in triplicates. To mimic the conditions present in the infection assay, the FCS concentration was reduced from 10 to 2%. 48 h after substance application, cell viability was measured with resazurin (10 µM concentration) at 485/20 590/20 nm for 2 h with the synergy plate reader. Pictures were taken to determine a visible cytotoxic effect. The percent viability was calculated using the slope of the increase in fluorescence in comparison to an untreated control with GraphPadPRISM version 9.0.0. Every experiment was independently conducted twice.

### Infection assay

Caco2 cells^11,54^ were seeded to a density of 40,000 cells/well in 48 well plates 48 h before infection, andVero-CCL81 were seeded at 30,000 cells/well, 24 h before infection. The substance dilutions were prepared in culture medium, either MEM (Gibco), supplemented with 1% penicillin–streptomycin (Sigma) and 2% FCS for Caco-2 cells, or Optipro medium supplemented with 1% penicillin–streptomycin and 1% L-glutamine for Vero CCL81 cells. Cells were then transferred to a biosafety level 3 facility (BSL-3), where they were incubated with the respective substance for 30 min. before addition of SARS-CoV-2 (Human 2019-nCoV ex China Strain: BavPat1/2020 Isolate: Germany ex China, Ref-SKU: 026V-038839) at a multiplicity of infection (MOI) of 0.825 (Caco-2) or 12 plaque-forming units (pfu) (Vero CCL81).The stock concentration was determined by TCID50. After 1 h of infection, the supernatant was removed, and cells were washed with medium 3 times. The cells were incubated at 37 °C and 5% CO_2_ for 48 h. Afterwards, the supernatant was harvested, and the virus was inactivated with QIAamp® Viral RNA Mini Kit Lysis buffer by Qiagen for later RNA isolation according to the manufacturer's protocol in a biosafety level 2 facility (BSL-2). The remaining supernatant was carefully removed to fix the cells with 4% buffered formalin for 20 min for immunohistochemistry staining. RNA samples were stored at − 70 °C until Quantitative Reverse Transcription PCR (qRT-PCR) analysis. Every experiment was independently conducted twice.

### qRT-PCR analysis of viral RNA

The qRT-PCR was performed using the QuantiTect Multiplex RT-PCR Kit (Qiagen) in a 25 µL reaction on the Rotor Gene Q (Qiagen) according to the manufacturer's protocol. Amplification was performed for 30 min at 50 °C and 15 min at 95 °C, followed by 45 cycles (95 °C for 3 min and 55 °C for 30 s). The following probe and primer sets for detection of the N-protein gene of SARS-CoV-2 were used as listed by the CDC^55^.

- 2019-nCoV_N1-F Forward Primer 5′-GAC CCC AAA ATC AGC GAA AT-3′.
- 2019-nCoV_N1-R Reverse Primer 5′-TCT GGT TAC TGC CAG TTG AAT CTG-3′.
- 2019-nCoV_N1-P Probe 5′-FAM-ACC CCG CAT TAC GTT TGG TGG ACC-BHQ1-3′ FAM, BHQ-1.
- 2019-nCoV_N2-F Forward Primer 5′-TTA CAA ACA TTG GCC GCA AA-3′.
- 2019-nCoV_N2-R Reverse Primer 5′-GCG CGA CAT TCC GAA GAA-3′.
- 2019-nCoV_N2-P Probe 5′-FAM-ACA ATT TGC CCC CAG CGC TTC AG-BHQ1-3′.

Results were analyzed with Microsoft Excel and Graphpad Prism7 software.

### Immunohistochemistry

After cell fixation with 4% formalin for 20 min, the cells were transferred to an BSL-2 lab for immunohistochemistry staining. First, cells were washed with PBS and then were permeabilized with Triton X-100 (Sigma) for 10 min, followed by washing with PBS (3 times for 3 min in each washing step). Then, 3% H_2_O_2_ diluted in methanol was added for 30 min to block endogenous peroxidases, followed by the PBS washing step. Afterward the cells were incubated for 1 h with 100 µL of a 1:1000 dilution of primary antibody (SARS-CoV-2 (2019-nCoV) Nucleocapsid Antibody, Rabbit Mab, Sinobiological Cat: 40143-R019) in antibody diluent (REAL Antibody diluent, Agilent Technologies, Dako Cat: S202230_2). After a washing step, cells were treated with the secondary antibody (EnVision™ + Dual Link System HRP, Agilent Technologies, Dako Cat: K5007) for 30 min, followed again by washing. Then, the substrate (AEC substrate-Chromogen, Agilent Technologies, Dako, Cat: K346430-2) was added dropwise (2 drops) on the cells and incubated until infected cells appeared red (no longer than 3 min). The reaction was stopped by washing, and cells were kept in PBS until photo documentation.

### Flow cytometry

Caco2 cells were harvested by incubation in 1 × accutase for 10 min, counted, and centrifuged at 400×*g* for 5 min at room temperature before washing and resuspension in FACS buffer (PBS supplemented with 0.05% sodium azide and 0.5% bovine serum albumin). A total of 1 × 10^6^ cells was incubated in FACS buffer containing the primary antibodies for ACE2 (LSBio, LS-C344721, mAb, mah, 1:500) and TMPRSS2 (abcam, ab280567, mAb, rah,1:200) or IgG1 (Agilent Technologies, X0931, 1:1000) in case of the control for 30 min at 4 °C. The cells were then washed in 2 mL of FACS buffer, centrifuged at 400×*g* for 5 min, and incubated in FACS buffer containing the secondary antibodies Alexa Fluor 647 goat-anti-mouse IgG (Invitrogen, A21235, 1:700) and Alexa Fluor 488 goat-anti-rabbit (Invitrogen, A11034, 1:700) in the dark for 20 min at 4 °C. After washing and centrifugation, the cells were resuspended in 1.5 mL FACS buffer before adding 0.5 mL 4% PFA for 10 min for fixation. After another washing step, the cells were resuspended in 400 µL FACS buffer, transferred to FACS tubes, and kept on ice in the dark until further use. Flow cytometry was performed using a CytoFLEX S flow cytometer equipped with the CytExpert Software (both Beckman Coulter). A typical cell area was gated, and 10,000 single events per sample were acquired.

Paraphrasing was performed on some sentences using the ChatGPT language model (OpenAI). The original text was inputted into the model, and the resulting paraphrased text was compared to the original to ensure accuracy and preserve the intended meaning. Any necessary revisions were made by the authors.

OpenAI. ChatGPT, a large language model trained on the GPT-3.5 architecture. Accessed [30.03.2023]. Available from: https://openai.com/blog/chatgpt-3/.

## Supplementary Information

Supplementary Figure S1.

Supplementary Figure S4.

Supplementary Figure S3.

Supplementary Figure S4.

## Data Availability

The CavitOmiX plugin for Schrodinger’s PyMOL, a tool that allows analyzing protein cavities from any input structure, is freely available for download at https://innophore.com/cavitomix/ and https://pymolwiki.org/index.php/CavitOmiX. It enables the analysis of protein structures, Catalophore cavities, and binding sites using crystal structures and AI models from OpenFold (powered by NVIDIA’s BioNeMo service), DeepMind`s AlphaFold, and ESMFold by Meta. If required, additional supplementary data can be obtained from the authors on request.
