# Supplementary figures and images for: Identification and validation of fusidic acid and flufenamic acid as inhibitors of SARS-CoV-2 replication using DrugSolver CavitomiX
Source: Sci Rep. 2023 Jul 21;13:11783. doi: 10.1038/s41598-023-39071-z (PMC10362000; doi:10.1038/s41598-023-39071-z)

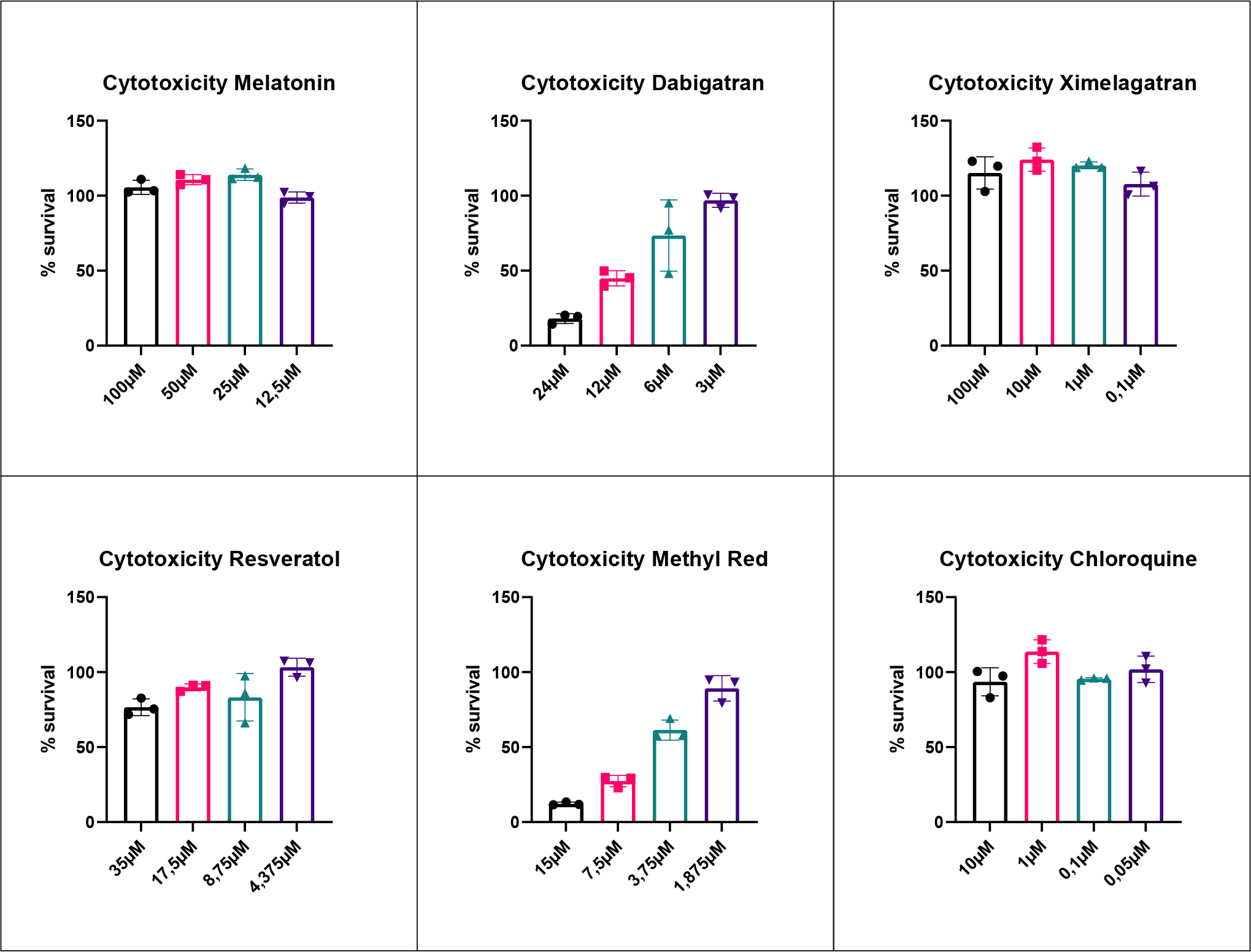

Supplement: Supplementary file 1 — Supplementary Figure S1. [file 41598_2023_39071_MOESM1_ESM.png]

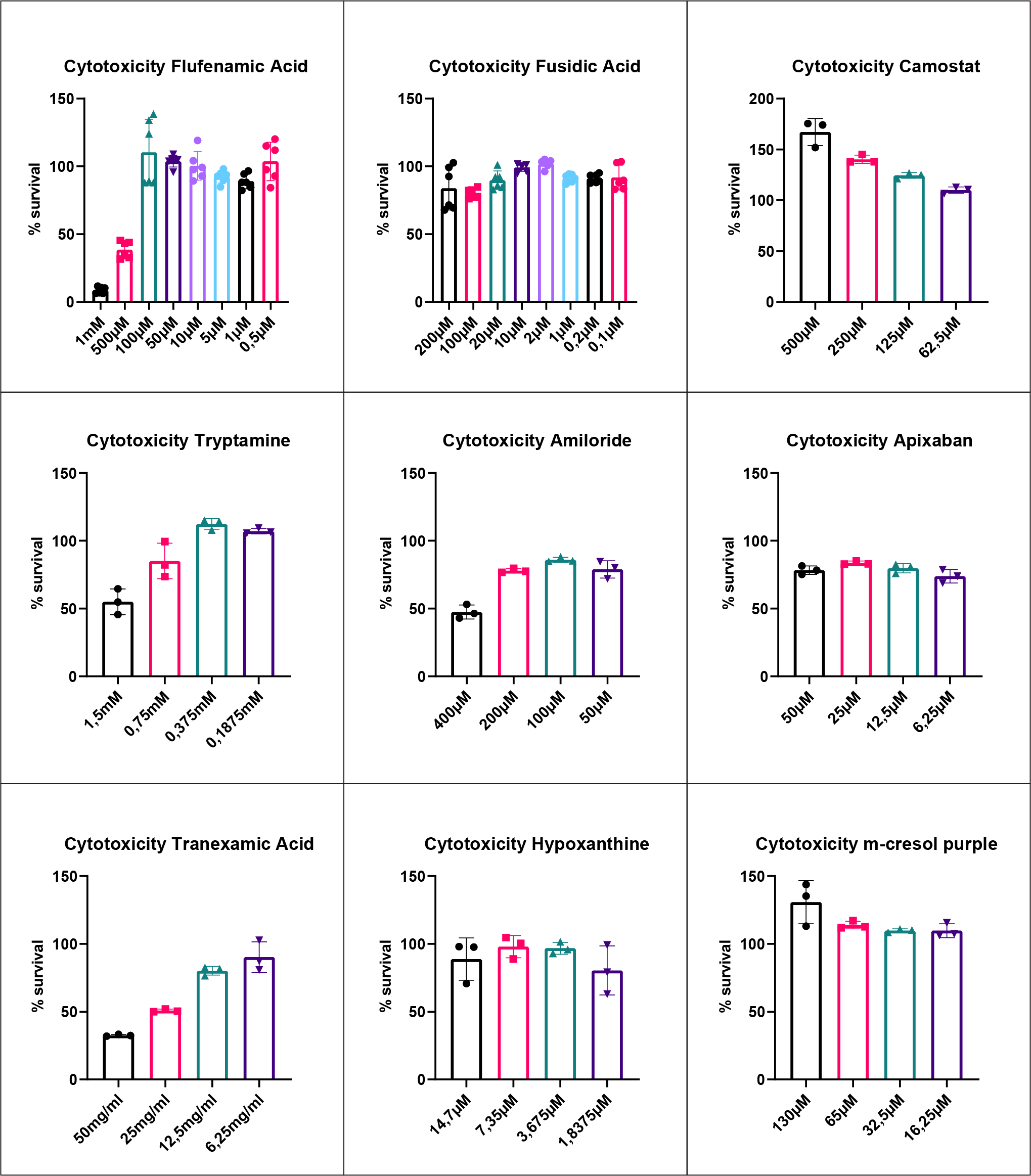

Supplement: Supplementary file 2 — Supplementary Figure S4. [file 41598_2023_39071_MOESM2_ESM.png]

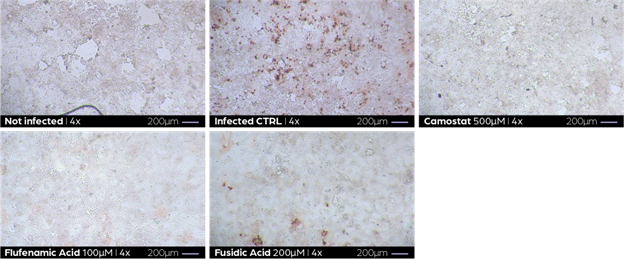

Supplement: Supplementary file 3 — Supplementary Figure S3. [file 41598_2023_39071_MOESM3_ESM.png]

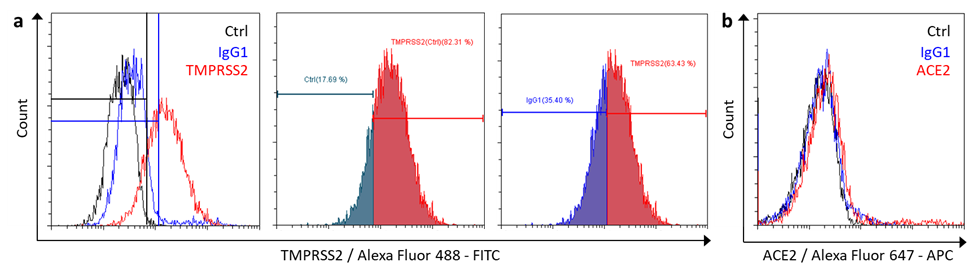

Supplement: Supplementary file 4 — Supplementary Figure S4. [file 41598_2023_39071_MOESM4_ESM.png]
